# Trajectories of Physical Activity Predict the Onset of Depressive Symptoms but Not Their Progression: A Prospective Cohort Study

**DOI:** 10.1155/2016/8947375

**Published:** 2016-10-04

**Authors:** Kaisa Kaseva, Tom Rosenström, Taina Hintsa, Laura Pulkki-Råback, Tuija Tammelin, Jari Lipsanen, Xiaolin Yang, Mirka Hintsanen, Christian Hakulinen, Katja Pahkala, Mirja Hirvensalo, Nina Hutri-Kähönen, Olli T. Raitakari, Liisa Keltikangas-Järvinen

**Affiliations:** ^1^Unit of Personality, Work and Health Psychology, Institute of Behavioral Sciences, P.O. Box 9, University of Helsinki, 00014 Helsinki, Finland; ^2^Helsinki Collegium for Advanced Studies, Fabianinkatu 24, P.O. Box 4, University of Helsinki, 00014 Helsinki, Finland; ^3^LIKES, Research Center for Sport and Health Sciences, Rautpohjankatu 8, 40700 Jyväskylä, Finland; ^4^Unit of Psychology, University of Oulu, P.O. Box 8000, 90014 Oulu, Finland; ^5^Research Centre of Applied and Preventive Cardiovascular Medicine, Kiinamyllynkatu 10, University of Turku, 20520 Turku, Finland; ^6^Paavo Nurmi Centre, Sports and Exercise Medicine Unit, Department of Physical Activity and Health, Kiinamyllynkatu 10, University of Turku, 20520 Turku, Finland; ^7^Department of Sport Sciences, P.O. Box 35 (L), University of Jyväskylä, 40014 Jyväskylä, Finland; ^8^Department of Pediatrics, P.O. Box 2000, University of Tampere and Tampere University Hospital, 33521 Tampere, Finland; ^9^Department of Clinical Physiology and Nuclear Medicine, Kiinamyllynkatu 4-8, Turku University Hospital, 20520 Turku, Finland

## Abstract

This prospective, community-based study examined trajectories of physical activity from childhood to adulthood and whether these trajectories contributed to depressive symptoms in adulthood to a greater degree than adulthood physical activity. Participants (*n* = 3596) were from the ongoing Cardiovascular Risk in Young Finns Study which started in 1980. Depressive symptoms were measured with Beck Depression Inventory (BDI-II) in 2012, and physical activity was assessed from 1980 to 2011 with self-reports. Analyses were adjusted for age, sex, childhood negative emotionality, socioeconomic factors, previous depressive symptoms, social support, body mass index, and smoking status (1980–2007). Highly, moderately, and lightly physically active trajectory groups were identified. Highly active participants reported lower levels of depressive symptoms compared to lightly active ones (*p* < 0.001) and compared to moderately active ones (*p* = 0.001). Moderately active participants had less symptoms than lightly active ones (*p* < 0.001). High levels of adulthood physical activity associated with lower levels of depressive symptoms (*p* < 0.001). The findings did not withstand adjustment for previous depressive symptoms (*p* > 0.05). Lifelong physical activity trajectories or adulthood physical activity was not associated with the progression of depressive symptoms in adulthood. Thus, physical activity history does not contribute to the progression of the depressive symptoms to a greater degree than adulthood physical activity.

## 1. Introduction

Depressive disorders affect 340 million people (approximately 9%) globally regardless of gender, culture, or ethnicity [1]. From 1990 till present time, depression has been rated one of the leading causes of years lived with disability (YLDs) [2]. Depression associates with a wide range of subsequent health outcomes. For instance, depression predicts future coronary events and cardiac deaths in healthy individuals and in those with established cardiovascular disease (CHD) [3, 4]. Depression has also been shown to be associated with several other physical diseases, including cancer and osteoporosis [4], as well as with severe psychiatric disorders such as schizophrenia [5]. Furthermore, depression relates to low educational attainment and reduced work productivity [6]. Identifying factors that contribute to depression and developing tools that can be applied in early prevention of depression have been prominently highlighted [7].

Even though some conflicting findings concerning the association between physical activity and depression exist [8, 9], prior evidence provides support for the value of physical activity in reducing depressive symptoms in both healthy and clinical populations [10]. Literature has also indicated that even low doses of physical activity may protect against depression [10]. The relation between physical activity and depression may also be bidirectional, as physical activity may alleviate depressive symptoms, but these symptoms may also decrease the likelihood of initiating physical activity [8, 11]. Physical activity has also been related to neurobiological functioning, which may preempt depressive mood [12]. In addition, physical activity has been shown to be associated with the development of individual qualities such as self-confidence, emotional self-regulation [13], and capacity of social bonding [14], which, in turn, may be protective against depression [13, 14].

Several studies have indicated that regular physical activity associates with reduced risk of depression in all age groups from early childhood to late adulthood [8, 10, 15]. There is also evidence that childhood physical activity may lead to maintenance of lifetime physical activity patterns conducive to well-being (e.g., [16]). These patterns, however, have a tendency to decline towards later adulthood [17]. Many previous findings regarding the association between physical activity and depression derive from cross-sectional designs [10]. Previous longitudinal or intervention designs cover relatively short follow-up phases of physical activity [10, 18]. Prospective studies examining the association between lifelong physical activity and depressive symptoms are very rare [8].

It has been stated that more sophisticated methods are needed when studying the development and etiology of depression [19]. It has also been shown that people's health behaviors may have various developmental tendencies instead of a one growth trajectory [20, 21], and some of these behavioral profiles may be more detrimental to health than others. For instance, it is theoretically possible that physical activity's decline towards later life [17] may start earlier and/or be steeper among specific groups than in others, and thus the different physical activity profiles may associate with distinct health outcomes. The need for examinations assessing the development and decline of physical activity has been acknowledged [17, 22]. It is also essential to study whether long-term physical activity contributes to health over and above the concurrent physical activity. Furthermore, identifying people, whose behavioral tendencies associate with decreased health, might be useful for professionals who aim to adjust lifestyle interventions to targeted groups. It is also important to gain information of the behavioral determinants and contributors of good health [23].

Growth mixture modeling provides an appropriate framework for studying developmental processes [20, 24]. The key advantage of such modeling is that it allows for the estimation of interindividual variability in intraindividual patterns of change over time [20, 24]. Current approaches to growth modeling have also been regarded as flexible for instance in terms of including partially missing data, unequally spaced measurement points, nonnormally distributed or discretely scaled repeated measures, and linearly or nonlinearly shaped trajectories [24]. These issues, or some of these, typically emerge in developmental research. It has been denoted that these issues present challenges for more traditional methods [24].

Extensive adjustment for potential confounders has been lacking in many previous studies regarding physical activity and health outcomes [16, 18]. Development of depression often depends on the interplay of multiple psychological and lifestyle related factors [7] that ideally should be considered when examining associations between health behaviors and depression [25–30]. Child's early emotional experiences (e.g., negative emotionality) may have effects on the development of depression later in life [25]. Childhood family's socioeconomic status has been shown to be associated with the development of health behaviors and well-being [26]. In adulthood, occurrence of depressive episodes has been shown to predict subsequent depression [27]. It has been proven that depression is related to age [28], and women tend to report higher levels of depression than men [28]. Adulthood socioeconomic position, experiences of social support, body mass index, and smoking status are also associated with depression in adulthood [26, 29, 30].

We examined the potential heterogeneity in physical activity trajectories in relation to depressive symptoms over a 30-year period from childhood to adulthood in a population-based sample with six cohorts and including eight study waves. To our knowledge, this is the first study assessing physical activity and depressive symptoms in such design. The analyses were adjusted for several risk factors for depression [25–30]. Physical activity measurements were performed during participants' childhood and adulthood (from the age of 9 to 49), and depressive symptoms were assessed in participants' adulthood (participants aged from 35 to 50). To gain a comprehensive picture of the association between physical activity and depressive symptoms, the association was studied cross-sectionally, with respect to change and with respect to long-term trajectories. The objectives of this study were (1) to explore the potentially distinct trajectories of physical activity from childhood to adulthood, (2) to examine whether physical activity was associated with cross-sectional, short-term, and long-term changes in depressive symptoms in adulthood, and (3) to examine whether the physical activity trajectories contributed to depressive symptoms in adulthood to a greater degree than adulthood physical activity.

## 2. Methods

### 2.1. Study Design and Participants

The study participants were from the ongoing prospective Cardiovascular Risk in Young Finns Study that began in 1980 [31]. The original sample consisted of 3596 children and adolescents (83.2% of those invited, 1832 females and 1764 males) from six birth cohorts (aged 3, 6, 9, 12, 15, and 18). To obtain a representative sample, Finland was divided into five areas based on the locations of universities with medical schools (Helsinki, Kuopio, Oulu, Tampere, and Turku), and the participants were randomly selected based on their social security numbers from nearby urban and rural areas. Informed consent was requested from each participant (or from the parents of small children), and the study was approved by the local ethics committees. The study was conducted according to Declaration of Helsinki and American Psychological Association's ethical principles.

The sample was followed in 8 waves, 1983, 1986, 1989, 1992, 1997, 2001, 2007-2008, and 2012, in which medical, psychological, and physical activity studies were conducted. Physical activity from childhood to middle adulthood was assessed in 1980, 1983, 1986, 1989, 1992, 2001, 2007, and 2011, response rate ranging from 53.1% to 72.8% (*n* = 1910–2619) of the original study participants (Table 1). Depressive symptoms were measured in 2012 and 47.9% (*n* = 1724) of the original study subjects participated in the examination (Table 1).

Previous studies of sample attrition have shown that there has not been systematic selection bias regarding study participants' medical profiles or physical activity [31, 32], but some selective attrition with respect to personality and depressive symptoms exists [33, 34]. Participants who were less self-directed and less agreeable and had higher levels of neuroticism as well as depressive symptoms had discontinued the study more often than others [33, 34].

### 2.2. Measures

#### 2.2.1. Physical Activity

Physical activity was assessed with self-administered questionnaires. Information concerning 3- and 6-year-old children's (born in 1974 and 1977) physical activity levels is missing from this study, because they were not able to self-report their physical activity levels in 1980. Participants, from whom physical activity was assessed, were aged from 9 to 18 in 1980 and from 34 to 49 in 2011.

From 1980 to 1989, physical activity questionnaires consisted of five questions focusing on the intensity and frequency of participants' leisure time physical activity, participation in sports-club training, participation in sports competitions, and participants' usual way of spending leisure time [35]. From 1992 till present time, questionnaires consisted of five questions as well, assessing the intensity and frequency of leisure time physical activity, hours spent on physical activity per week, average duration of a physical activity session, and participation in organized physical activity [32]. From 1980 to 1992, the answers for the questions were coded into 3 categories (ranging from 1 to 3), excluding the items that assessed participation in sports competitions (1980–1989) and participation in organized sports (1992) which had a response range from 1 to 2. From 2001 to 2011, all responses to the questions were coded into 3 categories (response scale ranging from 1 to 3). A sum score (physical activity index) of question responses was created for each participant each year (Table 1), higher scores reflecting higher physical activity level. The index has been found reliable and valid [32].

#### 2.2.2. Depressive Symptoms

Participants' depressive symptoms were assessed with Beck Depression Inventory II (BDI-II) [36]. These symptoms were measured in 2012 when the participants were aged from 35 to 50. BDI-II consists of 21 symptoms with a severity range from 0 (no symptoms) to 3 (severe level of depressive symptoms). A sum score of all items was computed for each participant (Table 1), and no missing items were allowed. The reliability estimate (Cronbach's *α*) for the depressive symptom scores was >0.90. BDI-II has demonstrated to be a valid instrument [36–38], and it has been regarded as an acknowledged standard in the measurement of depressive mood [36–39]. It is applicable in clinical and nonclinical contexts, including in general populations [36–39]. BDI-II correlates highly with its earlier versions, including modified BDI [36, 38], which has also been considered as a valid measure for assessing depressive symptoms in general populations [34]. Furthermore, BDI-II correlates well with other widely used scales for depression [38]. The instrument has been designed and also demonstrated to be a useful screening tool for potential depressed cases [36, 38].

#### 2.2.3. Covariates

Childhood, adulthood, and general (age, sex, and body mass index) covariates were controlled for in this study (Table 1) [25–30]. Participants' negative emotionality [25] was reported by the primary caretaker via six questions reflecting participants' behavior in childhood (e.g., “The child hits/kicks other children “accidentally””), on a scale from 1 (true) to 2 (not true), and average of the items was calculated for each participant. As some of the participants were adolescents in 1980, their caretakers responded to this question retrospectively. Participants' parents' socioeconomic status was assessed via two indices, educational and income levels [26]. Parents' educational level was determined via educational information collected from participants' mothers' and fathers' (1 = below 9 years/comprehensive school, 2 = 9 to 12 years/secondary school, and 3 = over 12 years/academic education). If parents' educational information differed, we based parental educational status on the information collected from the parent with the higher educational level. If educational information was available for only one parent, family's educational status was determined using his/her educational information. Family's income level was rated in an 8-point scale [1 =< 15 000 marks (~2523 euros) and 8 => 100 000 marks (~16819 euros)].

Symptoms of participants' adulthood depression were determined in 1992, 1997, 2001, and 2007 via a modified version of Beck Depression Inventory, referred to as a modified BDI [34, 40]. Items of the measure were rated in a 5-point scale, and average of the items was computed each year for each participant. Participants' socioeconomic status (2007) was determined via two indices; education was assessed via a 3-category scale (1 = comprehensive school, 2 = secondary school, and 3 = academic level) and income level via an 8-point scale (1 =< 10 000 euros and 8 => 70 000 euros). Additionally, participants' experiences of social support, body mass index, and smoking status measured in 2007 were controlled for [29, 30]. Social support was determined via a 12-question inventory [41] using a 5-point scale, and a mean score of the items was calculated for each participant. Participants' smoking status was examined via a 5-category scale (1 = smokes a cigarette per day or more, 2 = smokes once in a week, 3 = smokes less than once in a week, 4 = has quitted smoking, and 5 = has never smoked).

### 2.3. Statistical Analyses

Physical activity questionnaires, which were designed for children and adolescents (1980–1989) and adults (1992–2011) differed slightly in their content. To assure that the findings of the present study were based on changes in physical activity and not due to a measurement artifact, a confirmatory factor model was used to examine whether the physical activity indices consisting of five indicator variables had measurement and structural invariance over time [42, 43]. Weighted least squares means and variance adjusted (WLSMV) estimation was used for all analyses [43]. The goodness of fit for scalar invariance was assessed with comparative fit index (CFI), Tucker-Lewis index (TLI), and root-mean square error of approximation index (RMSEA). Factor scores derived from this examination were used in subsequent analyses.

Within the growth mixture modeling framework, Latent Class Growth Analysis (LCGA) was used to explore the trajectories of physical activity from childhood to adulthood. LCGA captures information about developmental processes at inter- and intraindividual levels, detecting subpopulations with distinct growth trajectories [20]. The determination of the number of subgroups for physical activity was based on Akaike's Information Criterion (AIC) [44]. In addition, the determination of the groups was based on the classification quality estimations and practical considerations [20, 45]. Within the LCGA model, the average temporal trajectories in the physical activity groups were modeled by regression equations, in which both the linear and quadratic terms were tested for the independent variable (time).

The associations between physical activity factor scores (assessed from age 9 to 49) and depressive symptoms (participants aged from 35 to 50) were first examined cross-sectionally and longitudinally with linear regression analyses. Due to the potential multiple testing problem, Bonferroni-corrected *p* values (*p* < 0.003) were used in determining the significant associations. Thereafter, the associations between the physical activity trajectory groups and depressive symptoms measured in 2012 were examined with analyses of variance, and post hoc tests were also performed (Bonferroni's method). Furthermore, we examined the longitudinal associations between physical activity levels assessed in participants' adulthood (2007, including participants aged from 30 to 45) and depressive symptoms (2012) using a linear regression. Due to the number of missing values, the variance analyses and the regression analyses in which the adulthood physical activity (2007) was used as a predictor were performed in another dataset which was imputed using the expectation-maximization (EM) algorithm [46]. Analyses were performed in statistical software programs Mplus (version 7.1 and version 7.2), IBM SPSS (version 21), and Stata (version 13). *p* values of <0.05 were considered significant.

## 3. Results

Descriptives of the original sample (*n* = 1724–3596) are shown in Table 1. Supplementary Tables 1 and 2 (see Supplementary Material available online at http://dx.doi.org/10.1155/2016/8947375) provide descriptives of the sample in the complete (*n* = 648) and imputed (*n* = 3564–3596) data, respectively. Although the scalar invariance model for physical activity did not demonstrate strong factorial invariance over time, the fit for partial scalar invariance model was adequate (CFI = 0.90, TLI = 0.90, and RMSEA = 0.047), given partial invariance of the threshold parameters. For RMSEA, values <0.05 indicate a very close model fit, and CFI and TLI values close to 0.90 denote an adequate fit [47]. Since the partial scalar invariance model was considered acceptable, factor scores were predicted for each subject to be used in subsequent analyses (Supplementary Table 3).

**Figure 1:**
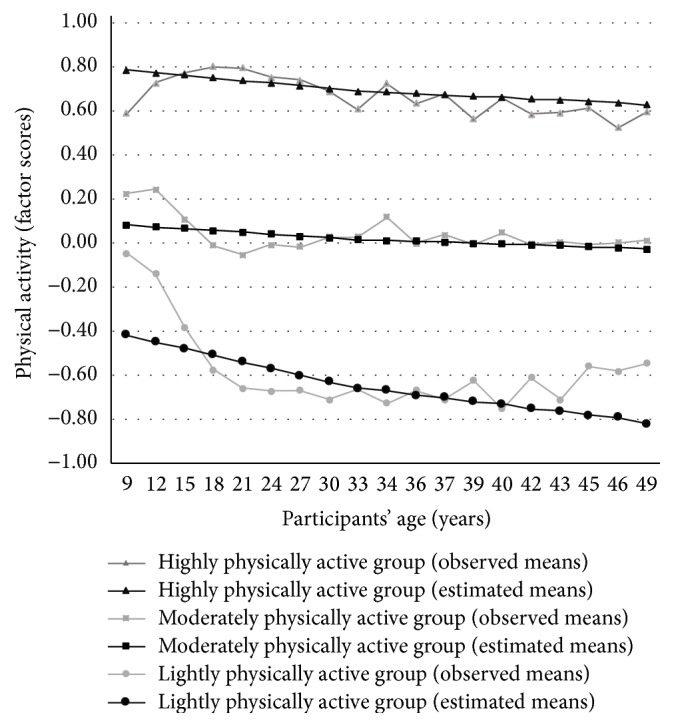
Means of the highly physically active (*n* = 147), moderately physically active (*n* = 3046), and lightly physically active (*n* = 371) trajectory groups from childhood to middle adulthood.

The prerequisites of growth mixture modeling (e.g., [24]) were met satisfactorily. LCGA suggested that a three-factor (group) solution was the best fitting model for the data based on AIC indices (AIC = 48915.44, 48915.39, 48901.43, 48902.85, and 48907.08 for 1, 2, 3, 4, and 5 groups, resp.; a lower AIC implies a better model). The classification quality of the three-factor model was also adequate based on the average probability estimates (group 1 = 0.74, group 2 = 0.86, and group 3 = 0.71). Values >0.70 delineate that the group consists of individuals with similar patterns of change [45]. Linear parameter estimates were found for the physical activity groups (Supplementary Table 4).  Figure 1 shows the central tendencies of the three groups, lightly (*n* = 371), moderately (*n* = 3046), and highly (*n* = 147) physically active groups by age. Based on the estimated marginal means, participants' physical activity levels remained relatively unchanged from childhood to adulthood in each group, although these levels appeared to diminish minimally towards middle adulthood in all participants (Figure 1).

The regression analyses performed in the original sample (*n* = 255–1467) indicated that low levels of physical activity factor scores were, in most examined ages, associated with higher levels of depressive symptoms in participants' adulthood (*p* < 0.05), although some of the associations attenuated when Bonferroni-corrected *p* values (*p* < 0.003) were applied (Table 2).

The analyses of variance in the original sample (*n* = 1722) indicated that the physical activity trajectory groups predicted symptoms of depression [*F* (2,1719 = 8.12, *p* < 0.001, adjusted *R* ^2^ = 0.01)]. Post hoc tests showed that highly physically active group had lower levels of depressive symptoms than lightly active group (mean difference = −3.26; *p* < 0.001, 95%CI: −5.25 to −1.26). Highly physically active participants had lower levels of depression in comparison to moderately active ones (mean difference = −1.92, *p* = 0.02, 95%CI: −3.66 to −0.18). Also moderately active participants had lower levels of depressive symptoms than lightly active participants (mean difference = −1.33, *p* = 0.02, 95%CI: −2.48 to −0.19). Based on the analyses of variance after adjusting for the covariates [25–30], the associations attenuated to nonsignificance [*F* (2, 631) = 2.13, *p* = 0.12, adjusted *R* ^2^ = 0.25]. In particular the previous symptoms of depression measured in 1992, 1997, 2001, and 2007 attenuated the associations (for details, see Supplementary Table 5).

**Figure 2:**
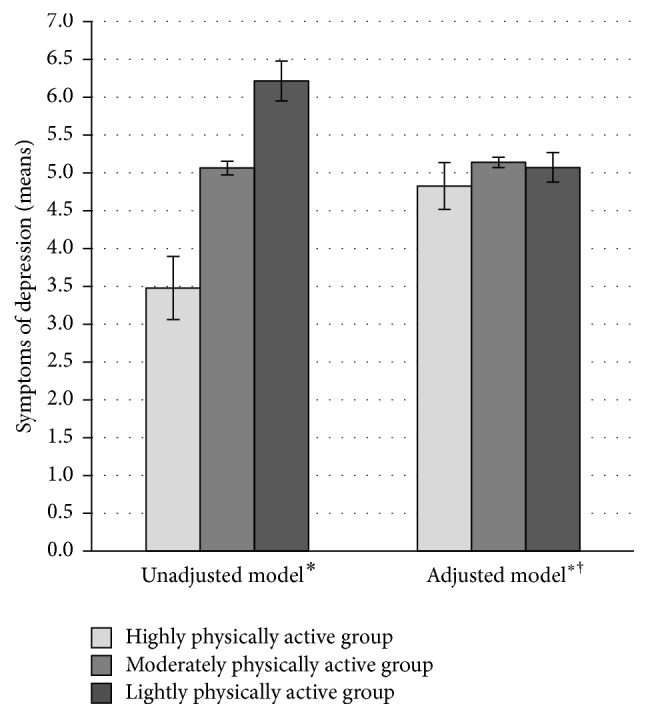
Symptoms of depression (2012) in the physical activity trajectory groups in unadjusted^*∗*^ and adjusted models^*∗*†^ (*n* = 3564). ^*∗*^Standard errors are represented in the figure by the error bars attached to each column. ^†^Participants' age, sex, childhood negative emotionality, parental education, parental income (1980), previous symptoms of depression (1992–2007), participants' education, income, social support, body mass index, and smoking status (2007) were adjusted for in the model.

We also examined the unadjusted associations in the data with full information on all study variables (*n* = 648), in which case the association between the physical activity trajectory groups and depressive symptoms became only marginally significant [*F* (2,645) = 2.76, *p* = 0.06, adjusted *R* ^2^ = 0.01]. As the sample attrition might have affected this association (i.e., by reducing the statistical power), the analyses were also performed in a dataset which was imputed using EM-algorithm (*n* = 3564–3596). This method was applied as the Little's MCAR test [48] confirmed that the data were not missing completely at random (*χ* ^2^ = 3903.02, *df* = 2923, *p* < 0.001). In the imputed dataset, physical activity groups predicted the symptoms of depression [*F* (2,3561) = 16.74, *p* < 0.001, adjusted *R* ^2^ = 0.01]. Post hoc tests indicated that highly physically active group had lower levels of depressive symptoms than lightly active group (mean difference = −2.74; *p* < 0.001, 95%CI: −3.91 to −1.56) (Figure 2). Highly physically active participants had lower levels of depressive symptoms in comparison to moderately active ones (mean difference = −1.59, *p* = 0.001, 95%CI: −2.60 to −0.57) (Figure 2). Also moderately active participants had lower levels of depressive symptoms than lightly active participants (mean difference = −1.15, *p* < 0.001, 95%CI: −1.81 to −0.49) (Figure 2). When the covariates [25–30] were adjusted for, the results became nonsignificant [*F* (2,3547) = 0.53, *p* = 0.59, adjusted *R* ^2^ = 0.47]. In particular the previous symptoms of depression measured in 1997 and 2001 attenuated the associations (for details, see Supplementary Table 5).

Thereafter, we examined the longitudinal associations between adulthood physical activity (assessed in 2007, participants' aged from 30 to 45) and symptoms of depression (assessed in 2012). In the original sample, adulthood physical activity was associated with depressive symptoms (*b* = −1.10, *p* < 0.001, 95%CI: −1.58 to −0.61, adjusted *R* ^2^ = 0.01). When the covariates [25–30] were adjusted for, the association attenuated to nonsignificance (*b* = −0.17, *p* = 0.62, 95%CI: −0.85 to 0.51, adjusted *R* ^2^ = 0.24). In particular the previous symptoms of depression assessed in 2001 and 2007 attenuated the association (for details, see Supplementary Table 6). Thereafter, the analyses were performed in a sample with full information on all study variables (*n* = 648), in which case the adulthood physical activity was not associated with depressive symptoms (*b* = −0.55, *p* = 0.16, 95%CI: −1.31 to 0.21, adjusted *R* ^2^ = 0.002). Due to the sample attrition, the association was studied also in the imputed data (*n* = 3596), and the results showed that the adulthood physical activity was associated with decreased levels of depressive symptoms (*b* = −1.07, *p* < 0.001, 95%CI: −1.36 to −0.78, adjusted *R* ^2^ = 0.01). When the covariates [25–30] were adjusted for, the association attenuated to nonsignificance (*b* = 0.06, *p* = 0.57, 95%CI: −0.16 to 0.28, adjusted *R* ^2^ = 0.47). In particular the symptoms of depression assessed in 2001 and 2007 attenuated the association (for details, see Supplementary Table 6).

## 4. Discussion

This study examined whether distinct trajectories of lifelong physical activity existed in the data and whether physical activity was related to depressive symptoms in adulthood. We also studied whether the lifelong physical activity trajectories contributed to the outcome to a greater degree than adulthood physical activity. This inspection was important, because the information whether lifelong trajectories contribute to depressive symptoms over and above the concurrent (adulthood) physical activity is lacking. LCGA revealed three distinct groups, the lightly, moderately, and highly physically active groups. Physical activity levels remained relatively similar from childhood to adulthood in each group, although the groups' physical activity levels decreased slightly towards middle adulthood. These results are in line with previous studies demonstrating the decline of physical activity with age [17].

High physical activity associated with lower level of depressive symptoms in adulthood, which is in accord with previous research [10, 18]. The mechanisms behind the observed association can be both physiological and psychological. Physical activity may alleviate depressive symptoms through neurobiological alterations [12]. Physical activity may also relate to enhanced self-confidence, emotion regulation skills, and social capacities that are associated with positive mood [13, 14].

The association between high physical activity and lower levels of depressive symptoms in adulthood, however, disappeared when covariates (age, sex, childhood negative emotionality, parents' socioeconomic status, participants' previous symptoms of depression, participants' socioeconomic status, social support, body mass index, and smoking status) were taken into account. The explanatory power of the fully adjusted models was substantially higher in comparison to the unadjusted ones (46% higher in the imputed data). These same results were found when lifelong physical activity trajectories and adulthood physical activity (assessed in 2007) were used as predictors for depressive symptoms in adulthood. Specifically, physical activity did not predict lower levels of depressive symptoms in adulthood when the experiences of previous depression were taken into account. The bidirectional nature of the association between physical activity and depression has been documented [8, 11]. Hence, it is theoretically possible that the childhood symptoms of depression preceding our first measurement have decreased the likelihood of initiating physical activity [8, 11] or that of maintaining adequate level of physical activity [11].

Summarizing, the study indicated that lifelong physical activity trajectories or adulthood physical activity levels were not associated with the progression of depressive symptoms in adulthood. The study suggests that lifelong physical activity history does not contribute to the progression of the depressive symptoms to a greater degree than adulthood physical activity.

### 4.1. Limitations and Strengths

Our results need to be interpreted in light of the following limitations. Participants did not provide information regarding each variable across the measurement years, which diminished the complete-sample size considerably. However, we attempted to control the potential attrition bias via an imputation method. The measurements concerning the participants' physical activity focused only on the self-reported leisure time physical activity, not total physical activity or energy expenditure. Also the depressive symptoms were assessed with self-administered questionnaires, and thus the possibility of subjective bias cannot be excluded. However, this is common in epidemiological studies by necessity. To our knowledge, comparably long follow-up studies using diagnostic interviews do not exist. Furthermore, early childhood depression was not assessed in the study, but this deficiency was retrieved to an extent by controlling for the childhood negative emotionality [25].

The strengths of the study were the prospective, population-based study design, relatively large sample size, use of LCGA, and utilization of an extensive set of covariates. In LCGA, the main advantage is that it allows a researcher to model developmental processes at inter- and intraindividual levels [20]. Such modeling has been shown to be especially useful regarding the development of health behaviors [20, 21], and the need for further studies has been recognized [22]. However, in the case of this study, the traditional cross-sectional and longitudinal studies yielded to very similar results. This was not evident* a priori*, and therefore our study adds evidence regarding the etiology of depression [19] by showing that physical activity trajectories appear not to play a special role over and above the adulthood physical activity.

Furthermore, depressive symptoms were studied with a well-validated instrument, BDI-II [36–39].

We were able to study the whole variance of participants' depressive symptoms instead of categorical diagnoses. This is important when attempting to find preventive tools for depression, because functional impairment is much more strongly associated with symptom severity than with diagnostic symptom count [49]. Also the people who have experienced symptoms of depression are at risk of getting a diagnosis [27].

### 4.2. Conclusions

This study identified three distinct physical activity groups, the lightly, moderately, and highly physically active ones. Each group's physical activity levels remained relatively unchanged from childhood to adulthood, although the levels tended to diminish slightly towards later adulthood. Physical activity was associated with depressive symptoms in adulthood. Highly physically active participants from childhood to adulthood had lower levels of depressive symptoms in adulthood compared to lightly physically active ones. Furthermore, participants' adulthood physical activity assessed in 2007 was associated with decreased levels of depressive symptoms in adulthood. The associations between physical activity and depressive symptoms disappeared when the preexisting symptoms of depression were controlled for, indicating that physical activities did not associate with the progression of depressive symptoms. Thus, the study suggests that lifelong physical activity history does not contribute to the progression of the depressive symptoms to a greater degree than adulthood physical activity. Obtaining information of mental health history might benefit clinicians and other professionals in evaluating the role of physical activity in well-being.

## Supplementary Material

The Supplementary Material consists of six tables.Supplementary Tables 1-3 present the descriptives of the data. Supplementary Table 4 presents the parameter estimates of the physical activity trajectory groups. Supplementary Tables 5-6 present the regression analyses between physical activity and symptoms of depression.

## Figures and Tables

**Table 1:** Descriptive statistics of the original sample (*n* = 1724–3596)^*∗*^.

Variables		Measurement year	*n*	Mean ± SD/%	Range
Covariates	Age	1980	3596	10.44 ± 4.99	3–18
	Childhood negative emotionality	1980	3177	1.06 ± 0.11	1–2
	Parental education^†^	1980	3540	1.90 ± 0.77	1–3
	Parental income	1980	3453	4.80 ± 1.94	1–8
	Symptoms of depression	1992	2330	2.14 ± 0.60	1–4.57
	Symptoms of depression	1997	2099	2.15 ± 0.67	1–4.57
	Symptoms of depression	2001	2097	2.07 ± 0.67	1–4.62
	Symptoms of depression	2007	2056	2.06 ± 0.68	1–4.67
	Participants' education^‡^	2007	2022	2.11 ± 0.90	1–3
	Participants' income	2007	2146	3.50 ± 1.56	1–8
	Social support	2007	2055	4.15 ± 0.80	1.08–5.00
	Body mass index	2007	2170	26.00 ± 4.75	16.56–58.82
	Smoking status	2007	2224	3.81 ± 1.53	1–5

Physical activity indices^§^	Physical activity	1980	2224	9.05 ± 1.83	5–14
	Physical activity	1983	2116	9.03 ± 1.88	5–14
	Physical activity	1986	2320	8.90 ± 2.01	5–14
	Physical activity	1989	2619	8.63 ± 2.10	5–14
	Physical activity	1992	2192	9.08 ± 1.92	5–14
	Physical activity	2001	2442	8.86 ± 1.96	5–15
	Physical activity	2007	2166	8.81 ± 1.81	5–15
	Physical activity	2011	1910	9.02 ± 1.88	5–15

Physical activity trajectory groups^§^	Lightly physically active	1980–2011	371	10.4%	
	Moderately physically active	1980–2011	3046	85.5%	
	Highly physically active	1980–2011	147	4.1%	

Dependent variable	Symptoms of depression (BDI-II)	2012	1724	5.04 ± 6.60	0–58

^*∗*^The original sample size was 3596, and 1764 (49.1%) of the participants were males and 1832 (50.9%) were females.

^†^The frequencies of parents' educational levels were as follows: low, *n* = 1228 (34.7%), average, *n* = 1428 (40.3%), high, and *n* = 884 (25.0%).

^‡^The frequencies of participants' educational levels were as follows: low, *n* = 713 (35.3%), average, *n* = 376 (18.6%), high, and *n* = 933 (46.1%).

^§^Physical activity indices ≤ 7 indicate low, >7 to 10 < moderate, and ≥10 high levels of physical activity. Factors scores, which were predicted from physical activity indices (1980–2011) (see Supplementary Table  3), were used in LCGA.

**Table 2:** Physical activity factor scores (assessed at participants' ages from 9 to 49) as predictors of symptoms of depression (participants aged from 35 to 50) (*n* = 255–1467).

Participants' age	*b*	SE	*Β*	*p* ^*∗*^	95% CI^†^
9	−1.06	0.51	−0.07	0.040	−2.06 to −0.05
12	−1.73	0.39	−0.13	<0.001	−2.49 to −0.98
15	−1.72	0.30	−0.15	<0.001	−2.30 to −1.14
18	−1.21	0.29	−0.11	<0.001	−1.78 to −0.63
21	−1.33	0.34	−0.11	<0.001	−1.99 to −0.66
24	−1.53	0.30	−0.15	<0.001	−2.12 to −0.95
27	−1.52	0.35	−0.15	<0.001	−2.21 to −0.84
30	−0.86	0.35	−0.08	0.015	−1.56 to −0.17
33	−1.16	0.47	−0.10	0.014	−2.08 to −0.24
34	−0.51	0.51	−0.06	0.324	−1.52 to 0.50
36	−0.68	0.49	−0.06	0.161	−1.64 to 0.27
37	−0.89	0.60	−0.09	0.140	−2.07 to 0.29
39	−1.59	0.44	−0.15	<0.001	−2.46 to −0.73
40	−0.34	0.72	−0.03	0.643	−1.76 to 1.09
42	−1.96	0.60	−0.18	0.001	−3.14 to −0.79
43	−1.65	0.61	−0.16	0.007	−2.85 to −0.45
45	−1.31	0.57	−0.13	0.022	−2.44 to −0.19
46	−2.22	0.58	−0.21	<0.001	−3.35 to −1.09
49	−1.34	0.54	−0.14	0.014	−2.41 to −0.27

^*∗*^Bonferroni-corrected *p* values (*α* = 0.05/19, *p* < 0.003) were used in determining significant associations.

^†^CI: confidence interval.
