# Supplementary material for: Trajectories of Physical Activity Predict the Onset of Depressive Symptoms but Not Their Progression: A Prospective Cohort Study
Source: J Sports Med (Hindawi Publ Corp). 2016 Oct 4;2016:8947375. doi: 10.1155/2016/8947375 (PMC5067320; doi:10.1155/2016/8947375)
Supplement: Supplementary file 1 — The Supplementary Material consists of six tables.Supplementary Tables 1-3 present the descriptives of the data. Supplementary Table 4 presents the parameter estimates of the physical activity trajectory groups. Supplementary Tables 5-6 present the regression analyses between physical activity and symptoms of depression. [file 8947375.f1.doc]

**Supplementary Table 1:** Descriptive statistics of the full sample (*n =* 648) ^*^.

| Variables |  | Measurement  year | Mean ± *SD*/  *n* (%) | Range |
| --- | --- | --- | --- | --- |
| Covariates | Age | 1980 | 10.47±4.97 | 3-18 |
|  | Childhood negative emotionality | 1980 | 1.04±0.10 | 1-2 |
|  | Parental education† | 1980 | 2.01±0.76 | 1-3 |
|  | Parental income | 1980 | 5.01±1.86 | 1-8 |
|  | Symptoms of depression | 1992 | 2.13±0.57 | 1-4.14 |
|  | Symptoms of depression | 1997 | 2.09±0.64 | 1-4.33 |
|  | Symptoms of depression | 2001 | 2.01±0.63 | 1-4.29 |
|  | Symptoms of depression | 2007 | 2.02±0.66 | 1-4.29 |
|  | Participants’ education^‡^ | 2007 | 2.21±0.87 | 1-3 |
|  | Participants’ income | 2007 | 3.55±1.39 | 1-8 |
|  | Social support | 2007 | 4.21±0.77 | 1.08-5.00 |
|  | Body mass index | 2007 | 25.61±4.52 | 16.56-45.89 |
|  | Smoking status | 2007 | 4.05±1.37 | 1-5 |
| Physical activity trajectory groups |  |  |  |  |
|  | Lightly physically active | 1980-2011 | 72 (11.1) |  |
|  | Moderately physically active | 1980-2011 | 536 (82.7) |  |
|  | Highly physically active | 1980-2011 | 40 (6.2) |  |
| Dependent variable | Symptoms of depression (BDI-II) | 2012 | 4.69±6.32 | 0-58 |

*648 Participants provided information regarding each study variable, and 413 (63.7%) were females and 235 (36.3%) were males.

^†^Frequencies of parents’ educational levels were as follows: low, *n* = 182 (28.1%), average, *n* = 274 (42.3%), high, *n* = 192 (29.6%).

^‡^Frequencies of participants’ educational status were as follows: low, *n* = 190 (29.3%), average, *n* = 129 (19.9%), high, *n* = 329

(50.8%).

**Supplementary Table 2:** Descriptive statistics of the imputed sample (for physical activity trajectory

groups, *n* = 3564, for other variables *n =* 3596).

| Variables |  | Measurement  year | Mean ± *SD*/  *n* (%) | Range |
| --- | --- | --- | --- | --- |
| Covariates | Age | 1980 | 10.44±4.99 | 3-18 |
|  | Childhood negative emotionality | 1980 | 1.06±0.11 | 1-2 |
|  | Parental education | 1980 | 1.90±0.76 | 0.82-3 |
|  | Parental income | 1980 | 4.78±1.91 | 1-8 |
|  | Symptoms of depression | 1992 | 2.16±0.52 | 1-4.57 |
|  | Symptoms of depression | 1997 | 2.14±0.56 | 1-4.57 |
|  | Symptoms of depression | 2001 | 2.08±0.57 | 1-4.62 |
|  | Symptoms of depression | 2007 | 2.07±0.57 | 1-4.67 |
|  | Participants’ education | 2007 | 2.06±0.71 | 1-3 |
|  | Participants’ income | 2007 | 3.45±1.25 | 1-8 |
|  | Social support | 2007 | 4.14±0.62 | 1.08-5.00 |
|  | Body mass index | 2007 | 26.03±3.75 | 16.56-58.82 |
|  | Smoking status | 2007 | 3.79±1.22 | 1-5 |
|  |  |  |  |  |
| Physical activity trajectory groups |  |  |  |  |
|  | Lightly physically active | 1980-2011 | 371 (10.4) |  |
|  | Moderately physically active | 1980-2011 | 3046 (85.5) |  |
|  | Highly physically active | 1980-2011 | 147 (4.1) |  |
| Dependent variable | Symptoms of depression (BDI-II) | 2012 | 5.12±5.04 | -1.95-58 |

**Supplementary Table 3:** Descriptive statistics of the physical

activity factor scores by age ( *n* = 537-3027) ^*^.

| Participants’  age | |  | | |
| --- | --- | --- | --- | --- |
|  | *n* | | Mean ± *SD* | Range |
| 9 | 1775 | | 0.23±0.45 | -1.27 to 1.67 |
| 12 | 2426 | | 0.23±0.51 | -1.61 to 1.83 |
| 15 | 3027 | | 0.09±0.58 | -1.79 to 1.95 |
| 18 | 3007 | | -0.05±0.59 | -1.63 to 2.04 |
| 21 | 2435 | | -0.10±0.58 | -1.73 to 1.91 |
| 24 | 2346 | | -0.06±0.59 | -1.76 to 1.96 |
| 27 | 1710 | | -0.08±0.57 | -1.84 to 1.86 |
| 30 | 1740 | | -0.03±0.59 | -2.00 to 1.66 |
| 33 | 1223 | | -0.02±0.54 | -1.76 to 2.08 |
| 34 | 557 | | 0.09±0.56 | -1.74 to 1.50 |
| 36 | 1247 | | -0.06±0.55 | -1.90 to 1.75 |
| 37 | 572 | | 0.00±0.55 | -1.76 to 2.00 |
| 39 | 1188 | | -0.09±0.56 | -1.87 to 1.53 |
| 40 | 646 | | -0.01±0.57 | -1.89 to 1.58 |
| 42 | 601 | | -0.08±0.57 | -1.92 to 1.87 |
| 43 | 651 | | -0.07±0.57 | -1.91 to 1.71 |
| 45 | 537 | | -0.09±0.60 | -1.88 to 2.25 |
| 46 | 601 | | -0.07±0.56 | -1.91 to 1.78 |
| 49 | 537 | | -0.08±0.61 | -1.96 to 2.13 |

^*^In 1980, participants were aged 9-18; in 1983, 9-21; in 1986, 9-24; in 1989,

12-27; in 1992, 15-30; in 2001, 24-39; in 2007, 30-45; and in 2011, 34-49.

**Supplementary Table 4:** Parameter estimates for the physical activity trajectory groups (*n* = 3564).

|  | Lightly physically active group | | Moderately physically active group | | Highly physically  active group | |
| --- | --- | --- | --- | --- | --- | --- |
|  | *β (SE)* | *p* | *β (SE)* | *p* | *β (SE)* | *p* |
| Intercept | -0.63 (0.05) | <0.001 | 0.03 (0.02) | 0.285 | 0.71 (0.07) | <0.001 |
| Linear* | -0.01 (0.001) | <0.001 | -0.00 (0.00) | < 0.001 | -0.00 (0.00) | <0.001 |

^*^The coefficient *β* represents the amount of increase or decrease in the dependent variable (physical activity) as the independent

variable (time) increases one unit.

**Supplementary Table 5:** Physical activity trajectory groups as predictors of depressive symptoms (2012) in the original (*n* = 1325-1722) and imputed (*n* = 3564)

samples controlling for each covariate separately in the models.

| Independent variable | Covariate | Measurement  year | Original sample* | Imputed sample† |
| --- | --- | --- | --- | --- |
| Physical activity trajectory groups | Age | 1980 | *F*(2,1718=7.80, *p*<0.001), *R^2^***=**0.01 | *F*(2,3560=15.39, *p*<0.001), *R^2^*=0.01 |
|  | Sex | 1980 | *F*(2,1718=7.56, *p*=0.001), *R^2^*=0.01 | *F*(2,3560=15.56, *p*<0.001), *R^2^*=0.01 |
|  | Childhood negative emotionality | 1980 | *F*(2,1536=6.67, *p*=0.001), *R^2^*=0.01 | *F*(2,3560=16.18, *p*<0.001), *R^2^*=0.01 |
|  | Parental education | 1980 | *F*(2,1692=6.33, *p*=0.002), *R^2^*=0.01 | *F*(2,3560=15.60, *p*<0.001), *R^2^*=0.01 |
|  | Parental income | 1980 | *F*(2,1664=5.56, *p*=0.004), *R^2^*=0.01 | *F*(2,3560=14.29, *p*<0.001), *R^2^*=0.02 |
|  | Symptoms of depression | 1992 | *F*(2,1334=2.44, *p*=0.088), *R^2^*=0.13 | *F*(2,3560=4.70, *p*=0.009), *R^2^*=0.23 |
|  | Symptoms of depression | 1997 | *F*(2,1336=2.33, *p*=0.098), *R^2^*=0.17 | *F*(2,3560=1.98, *p*=0.139), *R^2^*=0.29 |
|  | Symptoms of depression | 2001 | *F*(2,1403=1.06, *p*=0.347), *R^2^*=0.21 | *F*(2,3560=0.64, *p*=0.528), *R^2^*=0.34 |
|  | Symptoms of depression | 2007 | *F*(2,1476=1.62, *p*=0.197), *R^2^*=0.31 | *F*(2,3560=3.46, *p*=0.032), *R^2^*=0.44 |
|  | Participants’ education | 2007 | *F*(2,1321=5.33, *p*=0.005), *R^2^*=0.01 | *F*(2,3560=10.54, *p*<0.001), *R^2^*=0.02 |
|  | Participants’ income | 2007 | *F*(2,1405=4.03, *p*=0.018), *R^2^*=0.02 | *F*(2,3560=10.78, *p*<0.001), *R^2^*=0.05 |
|  | Social support | 2007 | *F*(2,1476=4.78, *p*=0.009), *R^2^*=0.07 | *F*(2,3560=9.88, *p*<0.001), *R^2^*=0.11 |
|  | Body mass index | 2007 | *F*(2,1405=5.91, *p*=0.003), *R^2^*=0.03 | *F*(2,3560=13.90, *p*<0.001), *R^2^*=0.04 |
|  | Smoking status | 2007 | *F*(2,1447=5.63, *p*=0.004), *R^2^*=0.01 | *F*(2,3560=14.00, *p*<0.001), *R^2^*=0.01 |

^*^In the original sample, previous symptoms of depression (1992-2007) attenuated all the associations between physical activity groups and depressive symptoms (2012).

^†^In the imputed sample, previous symptoms of depression (1997 and 2001) attenuated all the associations between physical activity groups and depressive symptoms (2012).

**Supplementary Table 6:** Adulthood physical activity (assessed in 2007, participants’ aged 30-45) as a predictor of depressive symptoms (2012) in the original

(*n* = 1325-1722) and imputed (*n* = 3564) samples controlling for each covariate separately in the models.

| Independent variable | Covariate | Measurement  year | Original sample* | Imputed sample† |
| --- | --- | --- | --- | --- |
| Physical activity (2007) | Age | 1980 | *b*=-1.09, *p*<0.001, *R^2^***=**0.01 | *b*=-1.04, *p*<0.001, *R^2^*=0.02 |
|  | Sex | 1980 | *b*=-1.10, *p*<0.001, *R^2^*=0.02 | *b*=-1.06, *p*<0.001, *R^2^*=0.02 |
|  | Childhood negative emotionality | 1980 | *b*=-1.22, *p*<0.001, *R^2^*=0.02 | *b*=-1.05, *p*<0.001 *R^2^*=0.02 |
|  | Parental education | 1980 | *b*=-1.02, *p*<0.001, *R^2^*=0.01 | *b*=-1.04, *p*<0.001, *R^2^*=0.02 |
|  | Parental income | 1980 | *b*=-0.95, *p*<0.001, *R^2^*=0.01 | *b*=-1.00, *p*<0.001, *R^2^*=0.02 |
|  | Symptoms of depression | 1992 | *b*=-0.70, *p*=0.009, *R^2^*=0.13 | *b*=-0.60, *p*<0.001, *R^2^*=0.23 |
|  | Symptoms of depression | 1997 | *b*=-0.60, *p*=0.024, *R^2^*=0.18 | *b*=-0.46, *p<*0.001, *R^2^*=0.29 |
|  | Symptoms of depression | 2001 | *b*=-0.34, *p*=0.156, *R^2^*=0.21 | *b*=-0.21, *p*=0.080, *R^2^*=0.34 |
|  | Symptoms of depression | 2007 | *b*=-0.18, *p*=0.434, *R^2^*=0.30 | *b*=-0.14, *p*=0.222, *R^2^*=0.44 |
|  | Participants’ education | 2007 | *b*=-0.97, p<0.001, *R^2^*=0.01 | *b*=-0.86, *p*<0.001, *R^2^*=0.03 |
|  | Participants’ income | 2007 | *b*=-0.79, *p*=0.002, *R^2^*=0.02 | *b*=-0.88, *p*<0.001, *R^2^*=0.05 |
|  | Social support | 2007 | *b*=-0.85, *p*=0.001, *R^2^*=0.07 | *b*=-0.68, *p*<0.001, *R^2^*=0.11 |
|  | Body mass index | 2007 | *b*=-0.85, *p*=0.001, *R^2^*=0.03 | *b*=-0.89, *p*<0.001, *R^2^*=0.04 |
|  | Smoking status | 2007 | *b*=-0.89, *p*=0.001, *R^2^*=0.01 | *b*=-0.98, *p*<0.001, *R^2^*=0.02 |

^*^In the original sample, previous symptoms of depression (2001-2007) attenuated all the associations between physical activity groups and depressive symptoms (2012).

^†^In the imputed sample, previous symptoms of depression (2001-2007) attenuated all the associations between physical activity groups and depressive symptoms (2012).
